# A Call for Conceptual Clarity: “Emotion” as an Umbrella Term Did Not Work—Let’s Narrow It Down

**DOI:** 10.3390/brainsci15090929

**Published:** 2025-08-27

**Authors:** Peter Walla, Angelika Wolman, Georg Northoff

**Affiliations:** 1 Faculty of Psychology, Freud CanBeLab, Sigmund Freud Private University, Freudplatz 1, 1020 Vienna, Austria; 2 Faculty of Medicine, Sigmund Freud Private University, Freudplatz 3, 1020 Vienna, Austria; 3 School of Psychology, Newcastle University, University Drive, Callaghan, NSW 2308, Australia; 4 Center for Brain Disorders and Cognitive Sciences, School of Psychology, Shenzhen University, 3688 Nanhai Blvd, Nanshan, Shenzhen 518060, China; angelikawolman67@gmail.com; 5 Mind, Brain Imaging and Neuroethics Research Unit, University of Ottawa, Institute of Mental Health Research, Royal Ottawa Mental Health Centre, 1145 Carling Avenue, Ottawa, ON K1Z 7K4, Canada; georg.northoff@theroyal.ca

**Keywords:** affective processing, cognitive processing, feeling, emotion, mood, affect, motivation, non-conscious processing, consciousness, parsimony, cephalization, Occam’s razor

## Abstract

To cut a long story short, the term “emotion” is predominantly employed as a comprehensive designation, encompassing phenomena such as feelings, affective processing, experiences, expressions, and, on occasion, cognitive processes. This has given rise to a plethora of schools of thought that diverge in their inclusion of these phenomena, not to mention the discordance regarding what emotions belong to the so-called set of discrete emotions in the first place. This is a problem, because clear and operational definitions are paramount for ensuring the comparability of research findings across studies and also across different disciplines. In response to this disagreement, it is here proposed to simplify the definition of the term “emotion”, instead of using it as an umbrella term overarching an unclear set of multiple phenomena, which is exactly what left all of us uncertain about the question what an emotion actually is. From an etymological perspective, the simplest suggestion is to understand an emotion as behavior (from the Latin verb ‘emovere’, meaning to move out, and thus the noun ‘emotion’ meaning out-movement). This suggests that an emotion should not be understood as something felt, nor as a physiological reaction, or anything including cognition. Instead, emotions should be understood as behavioral outputs (not as information processing), with their connection to feelings being that they convey them. Consider fear, which should not be classified as an emotion, it should be understood as a feeling (fear is felt). The specific body posture, facial expression, and other behavioral manifestations resulting from muscle contractions should be classified as emotions with their purpose being to communicate the felt fear to conspecifics. The underlying causative basis for all that exists is affective processing (i.e., neural activity), and it provides evaluative information to support decision-making. The essence of this model is that if affective processing responds above a certain threshold, chemicals are released, which leads to a feeling (e.g., felt fear) if the respective organism is capable of conscious experience. Finally, the communication of these feelings to conspecifics is happening by emotion-behavior (i.e., emotions). In summary, affective processing guides behavior, and emotions communicate feelings. This perspective significantly simplifies the concept of an emotion and will prevent interchangeable use of emotion-related terms. Last but not least, according to the current model, emotions can also be produced voluntarily in order to feign a certain feeling, which is performed in various social settings. Applications of this model to various fields, including clinical psychology, show how beneficial it is.

## 1. Introduction

This paper has a theoretical nature; however, it is not primarily a review of existing models; it is, more so, highlighting an evident problem that has existed for a long time and it finally suggests a quite simple solution. Clearly, it is going to be a difficult endeavor, because multiple existing concepts of the meaning of an emotion are hardwired in human brains. However, the fact that the hard wirings differ between different schools of thought should convince at least serious scholars that change is needed and a common understanding is highly desired, if not essential, to further our understanding of the human psyche and the behavior it generates. Emotion research spans various disciplines, including psychology, neuroscience, and philosophy among many others. However, a significant obstacle to progress in this field is the lack of consistent and precise terminology. Various terms are often used interchangeably, or at least not well defined as separate things, leading to confusion and hindering meaningful comparisons across studies [1,2] (Fox, 2018; Adolphs et al., 2019). This paper highlights the need for greater conceptual clarity and proposes a framework (The ESCAPE-Model: **E**motion**S C**onvey **A**ffective **P**rocessing **E**ffects; or simply the Walla-Emotion-Model) for more rigorous and consistent terminology in emotion research (or better, in affective neuroscience).

Imagine a clinical psychologist proposing emotion regulation [3] (e.g., Renna et al., 2017) as the optimal treatment option for a client. Obviously, in order to design a therapy and further develop it, it would be a beneficial prerequisite to know what it is that is meant to be regulated. At present, different psychologists, and even different key players in emotion research, would explain the workings of the treatment in different ways. In the worst case, this could result in incorrect interpretations of treatment strategies and outcomes, and generate misunderstandings between professionals. This is considered a problem [4] (see Walla, 2018). In their 2013 publication, Walla and Panksepp [5] proposed the so-called car analogy to highlight the problem. The authors emphasized that the term “emotion” is employed in the extant literature as if one would designate the wheels of a car “car”, the engine “car”, the entire car “car”, and even driving would be called “car”. This is an example of an illogical use of language. Unfortunately, this problem applies to more or less all terminology in affective neuroscience (including interchangeable use of terms), and most importantly to the term “emotion”, but to various other related terms as well.

Interestingly, this problem is not new. In 1981, Kleinginna & Kleinginna [6] published an extensive review and categorization of emotion definitions, which they found by undertaking a comprehensive analysis of the existing literature on emotion. The result was an incredible number of 92 definitions, which they could put into no less than 11 categories, showing the great diversity of opinions on the subject. Their work highlighted the complexity of the concept of emotion, demonstrating that it encompasses a wide range of factors, including subjective experience, physiological responses, behavioral expressions, and even cognitive aspects like those already mentioned in the abstract. Their work provided a valuable overview of the landscape of emotion definitions, highlighting the challenges of defining this term. This dates back to 1981. The question arises, “Does the concept of “emotion” need to be understood as so complex?” This doubt receives meaning, because more than 20 years after Kleinginna & Kleinginna, Scherer (2005) [7] published an article that included the question “What are emotions?” in the title. The article highlights, again, the complex nature of defining and measuring emotions as well as the challenges in achieving a consensus on what constitutes an emotion, differentiating it from related affective phenomena like feelings, moods, attitudes, etc. Surprisingly, nothing has changed. The result of the author’s own view is a model of emotion, which involves synchronized changes in several subsystems in response to significant stimuli, which is, again, a quite complex approach to define the term emotion even including cognitive aspects. The shocking truth is that another 20 years have passed and, to date, in 2025, we still face a lack of a clear definition. It was in 2012 when Dixon [8] published a paper about the history of the term “emotion”. In this work, he mentioned that, given the missing scientific consensus, it might be that the “very category of emotion” is the problem. There definitely is time for change and the change needs to be radical. Key players in emotion research are all contributing a great deal of detail regarding neurophysiological processes, anatomical structures, the release of chemicals, experiences, and, also, treatment strategies. However, there is no common agreement on how to define the term “emotion” [9,10,11,12,13,14,15,16,17,18,19,20,21,22] (Simic et al., 2021; Barrett, 2017; LeDoux, 2012; Scherer, 2009; Izard, 2009; Barrett, 2006; Rolls, 2005; Panksepp, 2005; Damasio, 2004; Cabanac, 2002; Damasio, 1999; Panksepp, 1998; Damasio, 1994; Lazarus, 1991). As already mentioned above, in response to this continuous disagreement, the present paper proposes a novel approach, the Walla-Emotion-Model. The proposed approach is incredibly simple. Some may consider it overly simplistic, but it is important to note that simplicity is not necessarily a disadvantage, provided that all the necessary aspects are still included. Complexity leading to disagreement, miscommunication, and misinterpretation cannot really be any better.

At this stage, it seems reasonable to mention the principle of parsimony [23] (Sober, 1981), often associated with “Occam’s razor” [24,25] (Thorburn, 1918; Spade, 2019), which advocates for choosing the simplest explanation when faced with competing hypotheses. It is a fundamental concept that permeates various fields, particularly science and philosophy. Essentially, parsimony dictates that among equally adequate explanations, the one with the fewest assumptions should be preferred. This principle encourages us to avoid unnecessary complexity and largely goes back to William of Ockham (although the concept predates him), a 14th-century English Franciscan friar and philosopher. His principle, often paraphrased as “entities should not be multiplied beyond necessity,” is a cornerstone of parsimony. According to work published in 1984 [26] (Epstein), psychologists often violate this principle, particularly in attributing complex behavior to cognitive processes. The same author goes on to give a practical definition of parsimony, which emphasizes that parsimony is a heuristic, not an absolute law. Simpler explanations are preferable, but they are not always correct. In the end, the goal should be to find the best balance between simplicity and explanatory power. In the context of the current problem, we do not primarily talk about explaining something. Instead, simplicity is here referred to as defining the term emotion in the narrowest possible way, which makes it easier to distinguish it from other terms such as feeling and affective processing. Secondly, this will also support explanations of those terms and thus support any communication within emotion research and affective neuroscience (crucially also in clinical psychology).

Not surprisingly, this is all based on neurobiological grounds. To better understand where this is all coming from, it is considered beneficial to start with basic neurobiological thinking about the evolutionary trend towards the concentration of nervous tissue and sensory organs at the anterior (front) end of an organism, which is known as cephalization [27,28] (e.g., Ruppert et al., 2004; Exner and Routil, 1958). Cephalization results in a distinct head region with enhanced sensory processing, improved coordination, and more complex information processing. Primarily observed in bilaterally symmetrical animals, cephalization facilitates directed movement and efficient interaction with the environment. Second, it is important to understand the human brain as an organ that processes information in order to produce adapted behavior (in addition to controlling all the vital functions to keep the whole organism alive) [4] (Walla, 2018). The next chapter follows this line of thinking while providing deeper explanations with a final focus on affective information processing in the brain (before cognitive information processing evolved).

## 2. From Cephalization to Affective Processing

In the primordial milieu, characterized by the emergence of diverse life forms, cephalization (the genesis of a head with a brain in it) occurred as a pivotal evolutionary development. Imagine, among mainly solitary forms of life stuck to a substrate, a diminutive, worm-like creature suddenly capable of simple locomotion, its nervous system comprising a rudimentary network, exhibiting a notable advantage due to the clustering of its neurons at its anterior end, where sensory tendrils interacted with the surrounding environment that varied as the worm moved along. The neurons located posterior to these sensors gathered right behind and were responsible for processing the sensory input, and the formation of a rudimentary “head” began, thereby establishing a centralized nervous system in it that updated the organism on external stimuli. This development led to enhanced directed movement, faster responses to danger, and an improved hunting efficiency. In summary, one can say that the occurrence of locomotion triggered the generation of a head with a simple brain in it [27] (Ruppert et al., 2004).

The continuous update on external stimuli formed a neural system behind the sensors that enabled an evaluation of the environment, which proved advantageous. The initial response manifested as a twitch rather than a thought, because neither cognition nor consciousness existed yet. Imagine the above-mentioned worm-like creature encountering a chemical gradient, with one direction representing a sweet, life-sustaining pulse, and the other, a bitter, corrosive sting. The neurons fired not with comprehension, but with a simple, binary “good” or “bad” response, an instinctual sorting process. It is evident that the ability to avoid bitter tastes and seek out sweet ones is advantageous for survival. Over successive generations, the neural knot at the front end underwent a process of growth and branching (simple to more evolved brain), leading to the establishment of more complex responses. These responses, sooner or later, involved the release of neurotransmitters and hormones (messenger chemicals that can modify neural responses) [29,30] (see Loewi, 1921; Jekely, 2021). A surge of dopamine, a primitive “wanting” response, drove the creature to seek out sweet tastes. Conversely, a surge of norepinephrine, a stress hormone, triggered a flight response, prompting the creature to avoid bitter substances.

These were not just signals anymore, they were drivers, shaping behavior and dictating survival. And as the brain grew, these drivers became more nuanced. Over the course of millions of years, the neural net underwent a transformation into a structured brain, capable of more than mere sensation and reaction; it began to evaluate in a more advanced way. The creature began to anticipate, which means that a faint echo of past experience began to color the present. The affective processing system was born [31,32,33] (Panksepp, 1991, 1992, 2011) and it was connected to a memory system. A cluster of interconnected structures, still situated deep within the modern brain, has emerged. The amygdala acts as a sentinel, scanning for threats with its neurons firing warnings [34,35,36] (Sergerie et al., 2008; Phelps et al., 2005; Zald, 2003). The hippocampus stores memories of past encounters, shaping future responses [37] (Squire, 1992). The hypothalamus orchestrates the body’s physiological reactions (neurotransmitters and hormones) as a regulator [38] (Purves et al., 2001). A proper affective information processing system has evolved, and it provides the organism carrying the brain, with this system in it, with processing output that helps its decision-making in order to adapt its produced behavior with respect to the question, “how is a stimulus”, and its answer.

According to the currently proposed model, this is all still completely separate to an emotion. In the next chapter though, the processes and constructs explained up until this point are amended by emotions.

## 3. From Affective Processing to Emotions

Further evolutionary developments resulted in the emergence of a social lifestyle with obvious advantages [39] (Wilson, 1975). In response to that, these internal affective states (chemical compositions) began to manifest externally, and a flick of the tail, a baring of the teeth, a vocalization—these were not conscious expressions of “anger” or “joy”, but rather, automatic, and behavioral responses. Those were observable and communicative. A predator, seeing bared teeth, understood the threat, and a mate, hearing the vocalization, understood the readiness. Over time, this primitive communication of internal states became more sophisticated. The neural structures that generated these states grew more complex, allowing for a wider range of expressions. With this, emotions as behavioral output arose to communicate inner states to conspecifics when language did not exist yet. The chemical composition in a brain representing threat was suddenly coupled with the outward display of trembling and widened eyes, which told others about the inner state. Such inner states were, at this point, not yet feelings, but important signals in the brain providing helpful contributions to survive that could now be shared among other members of the group.

## 4. From Affective Processing to Feelings and the Rise of Cognition

With the emergence of consciousness, those inner states became felt bodily responses to affective processing evaluations. Feelings were born. While the limbic system, the cradle of affection [40] (Papez, 1937), was not built for conscious thought, but for the visceral, instinctual imperatives of survival, consciousness arose in response to the rise in the most modern neural layer in the human brain, the neocortex. Although the affective processing system already allowed the brain to adapt its produced behavior to support survival, this further system evolved and was able to contribute other aspects of the ever-changing environment to decision-making processes. Those aspects are like answers to “what”-questions and the underlying function is what we know as cognitive information processing [4] (see Walla, 2018).

Cognition and affection are separate systems (as outlined in more detail below). The combination of affection and consciousness creates feelings and the existence of cognition can turn a raw “want” to desire, and raw “fear” can become anxiety. However, according to the current model, affection and cognition are separate systems with their different contributions to the overall function of the brain to produce adapted behavior (see next chapter, “Section 5”).

Finally, the evolution of language [41] (Pinker & Bloom, 1990), the pinnacle of cephalization, allowed for the most complex form of communicated feelings. Humans, their brains overflowing with neural connections, could not only feel fear and show it through generated emotion-behavior, but describe it, analyze it, and share it verbally. The internal world, once a hidden landscape of chemical signals, was now a shared territory of conscious experience, a testament to the long journey from a simple head to a mind capable of feeling and telling the story of those feelings. However, at this stage it is important to mention that the phenomenon known as “cognitive pollution” [42] (Walla et al., 2011); this is the potential of being led to subjective misinterpretations when asked to verbalize a feeling. This might be important to Clinical Psychology and Psychotherapy.

## 5. The Function of the Brain

**Figure 1:**
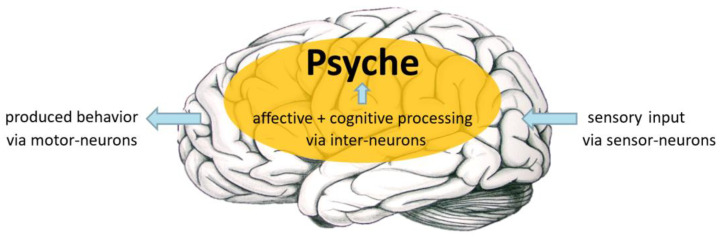
Besides controlling vital body-physiology, the brain’s function is to produce adapted behavior. Adaptation happens via processing sensory input (delivered by sensory neurons) and feeding the results to decision-making neural networks (interneurons) and, finally, initiating the execution of respective behavior (via motor neurons). It is proposed that the sum of information processing leading to executed behavior equals the “psyche” and is a composition of affective and cognitive processing. Affective processing is considered to provide evaluative outcome (good or bad; pleasant or unpleasant), while cognitive processing is considered to provide semantic outcome (what is it). The figure is taken and adapted from [43] Walla (2023).

The shortest possible explanation of the brain’s function is that it produces adapted behavior via processing information (as mentioned above, this is in addition to its basic function as a control center to keep the entire organism alive). Thereby, information equals voltage-coded signals that represent affective content (answers to how-questions) and cognitive content (answers to what-questions) [4] (see Walla, 2018). Importantly, those two processing systems are separate neural networks, including their own neural structures (see above). However, there are numerous connections that enable interactions and mutual influence. Figure 1 visualizes the brain’s function.

The neurobiological basis explained above finally leads to the proposed emotion-model, which is highlighted in the following chapter.

## 6. The ESCAPE-Model (EmotionS Convey Affective Processing Effects) (The Walla-Emotion-Model)

**Figure 2:**
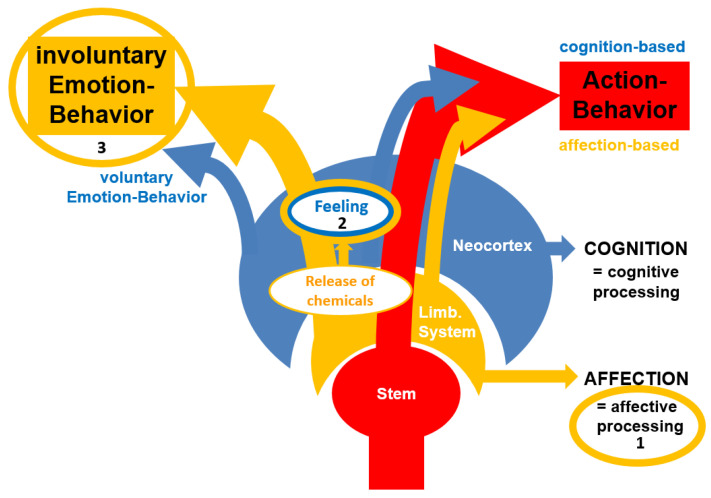
Emotions convey affective processing effects—Model (ESCAPE-Model) (or Walla-Emotion-Model). In short, it means that (**1**) affective processing guides behavior by providing evaluative information to support decision-making for the production of action-behavior. (**2**) Supra-threshold affective processing leads to released chemicals (Neurotransmitters and hormones), which are felt by an organism that is capable of conscious experience. (**3**) Involuntary emotion-behavior is produced to communicate a felt state to conspecifics.

This model (Figure 2) contrasts action-behavior with emotion-behavior. It is a further-developed model initially reported by Walla (2018) [4]. Action-behavior is basically initiated by brainstem networks (thick red arrow pointing to the right) and, on the way to producing action-behavior, both affection (orange-colored limbic system) and cognition (blue-colored neocortex) provide their adaptation-related input. This could be compared with the so-called survival circuits reported by LeDoux [11] (2012). If the limbic system (i.e., affective processing) responds to evaluated stimuli with activity above a certain threshold, it causes the release of chemical substances (neurotransmitters and hormones), a phenomenon that can be felt if the respective organism is capable of consciousness. Thus, a feeling as such is a form of perception, which in turn is a construct of the psyche [44,45,46] (see Helmholtz, 1910; Gregory, 1997; Sternberg, 2006). In contrast to Lisa Barrett’s constructed emotion theory [47] (2017), our model defines only a feeling as a construct of the psyche. Anyway, independent from felt released chemicals, emotion-behavior is produced. While in principle emotion-behavior is produced involuntarily, humans can also produce voluntary emotion-behavior, which is often performed in various social settings, where actual feelings are hidden with a certain cognitive purpose.

Because of its simplicity, this model is able to provide very short explanations of the most important terms around the topic of emotion. The very basis for an “emotion” is affective processing (Figure 2; orange circle 1), which is understood as neural activity of structures that are part of the limbic system. Affective processing delivers evaluative information to decision-making centers. If those structures become active above a certain threshold, neurotransmitters and hormones are released, which is felt by organisms that are capable of consciousness. This causes feelings (Figure 2; orange circle 2). Finally, emotion-behavior (Figure 2; orange circle 3) is produced in order to communicate feelings.

## 7. Discussion

As mentioned in the introduction, the motivation for this paper is the problem of a missing consensus regarding a clear definition of the term emotion. Over many decades (even centuries), the term “emotion” has notoriously been defined in various different ways. Across the history of psychology and neuroscience, various prominent researchers contributed valuable and highly appreciated insights into different aspects around affective processing and the entire field of emotion research. However, they have approached this topic from different angles, emphasizing distinct components or functions and creating different definitions, and the resulting missing consensus hinders any progress and makes scientific communication almost impossible.

To name just a few existing views, for instance, the core idea of Charles Darwin’s evolutionary perspective on emotions is that they are characterized by their observable expressions and their adaptive value [48] (Darwin, 1872). Darwin did not specifically define the term emotion as such, but one can infer that, despite the emphasized expression-related aspect of emotions, they were seen as instinctive states functioning as universal communicative signals across species and cultures, aiding in survival and social interaction. Interestingly, Darwin put less focus on the subjective feeling aspect, which might result in his perspective being closest to the proposed definition of emotion in the current paper. Most psychology textbooks mention William James [49] (James, 1884) and Carl Lange [50] (Lange, 1912), who are known for their physiological Feedback Theory, which emphasizes that our bodily responses precede and cause the conscious feeling of an emotion. Obviously, their understanding of an emotion is that it is a felt perception of physiological changes in the body. In summary, their view means that one does not cry because of being sad but, rather, one feels sad because of crying. The problem here is that the terms emotion and feeling are not clearly distinguished; instead, both authors speak about “felt emotions”. Equally often in textbooks one finds Walter Cannon [51] (1927) and Philip Bard [52] (1928), who proposed the so-called Thalamic Theory of Emotion, which challenged the theory proposed by James and Lange. Their core idea was that physiological responses and the conscious emotional experience occur simultaneously and independently, triggered by the thalamus, a diencephalic brain structure directly connected to the hypothalamus, which is heavily involved in the release of chemical substances. In fact, it is difficult to extract a clear definition of the term emotion in their case. However, one can summarize their view on emotion as involving parallel processing, where a stimulus leads to both a bodily response and a subjective emotional experience via brain activity. Also, here, an emotion is a felt phenomenon associated with both physiological and subjective aspects. Schachter and Singer [53] (1962), as well as Schachter [54] (1964), are known for their Two-Factor Theory, which integrates elements of James–Lange and Cannon–Bard, positing that emotion is a combination of physiological arousal and cognitive appraisal. Their understanding of an emotion is that it is a cognitive interpretation of a general physiological arousal based on the context of the situation. The same arousal (e.g., racing heart) can be interpreted as fear, excitement, or anger depending on the situation. Thereby, the crucial role of cognitive interpretation is in labeling and shaping the emotional experience. Again, an emotion is understood as something felt. Andrew Ortony, Gerald Clore, and Allan Collins [55] (1988) developed the known OCC Model of emotion, which represents a Cognitive Appraisal Theory. The basic idea of this model is that an emotion is a primarily valenced reaction to an event, an agent, or an object, with their particular nature being determined by the way in which the eliciting situation is construed (appraised). Strikingly, an individual’s interpretation or evaluation of a situation (their appraisals) directly determines the specific emotion experienced. In summary, this model categorizes emotions based on the focus of these appraisals (e.g., events, agents, and objects).

Paul Ekman [56,57] (1971, 1992) proposed a theory focusing on a set of discrete emotions that he suggested to be universal across human cultures and associated with distinct, innate facial expressions and physiological patterns. Emotions are thus understood as discrete, biologically programmed packages of responses (including facial expressions, physiological changes, and subjective experience) that serve adaptive functions. Edmund Rolls [58] (2025) understands emotions as states elicited by rewards and punishers, with different emotions corresponding to different types of reward/punishment contingencies (e.g., presence of reward, omission of reward, and presence of punisher). He emphasizes that the brain is organized to process these reinforcing stimuli to guide flexible, adaptive behavior linking emotions directly to motivation and goal-directed behavior. James Russell [59] (1980) is a proponent of dimensional models (like his Circumplex Model; core affect and prototypical emotional episodes). He argues that emotions are best understood as varying along continuous dimensions, primarily valence (pleasantness–unpleasantness) and arousal (high–low activation). Any specific emotion (e.g., anger) can be located as a point or region within this 2D space. He suggests that humans construct their emotional experiences based on learned concepts rather than emotions being hard-wired phenomena. His concept of a core affect is attributed to a specific cause and that it can be integrated with other cognitive and behavioral processes leading to labeled identifiable experiences. However, Russell often argues that the everyday concept of “emotion” is fuzzy and heterogeneous instead, and not a scientifically precise term. He suggests that relying too heavily on everyday language for scientific definitions can be misleading. Interestingly, as a matter of fact, there does not seem to be a large difference between everyday language and scientific language, because scientific language concerning the term emotion is equally non-specific as everyday language, which forms the main argument here to narrow-down the definition of the term emotion. A more comprehensive guide on existing emotion theories has recently been published [60] (Scarantino, 2024).

In summary, the varying definitions highlight a fundamental debate in emotion research that results in various groups of approaches. Regarding a nature versus nurture approach (i), Darwin, Ekman and Rolls are nature-oriented by emphasizing the innateness of emotions. Schachter and others, on the other hand, are rather nurture-oriented with a focus on learned and culturally influenced emotions. Following a distinction between discrete and dimensional views (ii), Ekman and Plutchik [57,61,62] (1958, 1962) define a few of distinct “basic” emotions, whereas, according to Russell’s opinion, emotions vary along continuous dimensions like valence and arousal. Despite these multi-faceted differences, there is consensus that emotions are multi-component phenomena involving subjective experience, physiological changes, cognitive appraisal, and behavioral/expressive elements. A crucial fact is that all approaches that understand emotion as an umbrella term struggle to find a common and agreed understanding, which results in emotion as a term that is not clearly defined. This shall be clarified, and instead of further hindering any clear scientific communication, and instead of further waiting until the set of components that should in combination represent an emotion is agreed on, it is here proposed to make a clear cut and define emotion simply as behavioral output. It is suggested to strictly narrow-down the meaning of the term emotion and to make a sharp distinction between non-conscious affective processing (i.e., neural activity reflecting evaluations of processed information) that guides behavior and observable emotions (i.e., behavioral responses to affective processing outcome) that communicate feelings. An emotion should be understood as an observable behavioral response that communicates an individual’s inner (feeling) state. Strikingly, the communicative function of emotions is emphasized, and emotions are clearly separated from underlying non-conscious processing (affection) and subjective feeling, and they are, finally, also separated from cognitive information processing.

Following the herewith proposed emotion-model has a great variety of positive consequences, some of which are mentioned below.

### 7.1. Key Consequences and Implications

The proposed, precise distinctions between affective processing (non-conscious neural activity guiding behavior), feelings (consciously felt bodily responses such as released neurotransmitters and hormones), and emotions (observable communicative behaviors) have significant consequences for clinical psychology and other applied fields. The current model emphasizes limitations of self-report and the importance of objective, neuroscientific measures to understand the true drivers of behavior (conscious free will is most likely rather limited regarding the guidance of human behavior).

#### 7.1.1. Clinical Psychology

The current model means that emotions are less important to investigate; the focus is more on affective processing and, if at all, on verbal communication of feelings. Because feelings represent conscious perceptions, the current model highlights that explicit self-reports of feelings can be “cognitively polluted” (a theory stated by Walla et al., 2011 [42]) and may not accurately reflect underlying, non-conscious affective processing that might be most directly related to psychological, affective disorders. Relying solely on questionnaires or verbal descriptions in clinical assessment might be misleading. For conditions where affective processing is impaired (e.g., depression, anxiety disorders, alexithymia, PTSD), objective measures like electroencephalography (EEG), magnetencephalography (MEG), skin conductance response (SCR), and startle reflex modulation (SRM) become crucial. These methods can access “raw affective responses” (particularly SRM) that are not directly accessible to language or conscious awareness [63,64,65,66,67,68] (Guo et al., 2022; Grillon & Baas, 2003; Morgan et al., 2003; Grillon &v Morgan, 1999; Patrick et al., 1993; Bradley et al., 1993). Furthermore, understanding discrepancies between what a patient reports about a feeling and what objective physiological or neural measures indicate could be key to understanding their condition. A feeling is a form of perception and perception is just a construct of the human psyche, a construct that can be wrong. For example, a patient might verbally deny feeling anxiety, but their physiological responses might tell a different story, revealing subconscious fear or distress. For individuals with alexithymia (i.e., difficulty identifying and describing what is usually labeled as emotions and here suggested to be called affections), this model provides a framework to understand this disorder as a disconnect between underlying affective processing and the ability to consciously label or communicate these states as adequate feelings. Objective measures of affective processing could be vital for diagnosis and tracking progress.

If affective processing primarily guides behavior at a basic level, interventions might need to move beyond purely cognitive–behavioral approaches to also address non-conscious affective learning and conditioning. Techniques like exposure therapy, which can modify non-conscious fear responses, align well with this. Therapists might need to differentiate between helping patients manage their observable “emotions” (e.g., reducing aggressive outbursts) versus helping them understand and regulate their underlying “affective processing”. The therapeutic strategy to regulate emotions could be well explained. Finally, certain neurological conditions might involve specific deficits in affective processing that are distinct from cognitive impairments. This opens avenues for early detection or targeted therapies and, of course, makes respective communication much more detailed and understandable. For example, Borderline Personality Disorder (BPD) is characterized by pervasive instability in mood, interpersonal relationships, self-image, and behavior [69,70] (APA, 2022; WHO, 2019). When one applies this paper’s precise definitions of affective processing, feelings, and emotions to BPD, we can gain a more nuanced understanding of the disorder’s core features, particularly its pervasive “affective” dysregulation. First, at the most fundamental level, individuals with BPD are theorized to have a biological vulnerability that leads to dysregulated or hyper-reactive affective processing. This means their subcortical brain system, including the amygdala among various other structures that are responsible for the initial, automatic evaluation of stimuli as threatening or rewarding, is often affected (see above chapter “Section 5”). Their affective processing can be hyper-sensitive. This means they detect and react to mainly negative stimuli more readily and intensely than neurotypical individuals, already at this completely non-conscious level. A subtle shift in a facial expression, or a slightly raised tone of voice can trigger an immediate, above-average neural response in the limbic system. So, for someone with BPD, their affective processing is like a highly sensitive and easily triggered alarm system, constantly on high alert, and slow to return to baseline. Second, the hyper-reactive affective processing directly translates into a cascade of neurotransmitter and hormone release, which in turn translates into intense, strong feelings. People with diagnosed BPD often struggle with the conscious cognitive process of accurately identifying, labeling, and understanding their feelings. This aligns with this paper’s idea to take out any cognitive aspect in the definition of the term emotion and its related terms. Any attempt to label (name) a feeling, even more so affective processing, is potentially affected by cognitive pollution [42] (see Walla et al., 2011). Third, the hyper-reactive neural responses, driven by dysregulated affective processing, manifest as problematic emotions, which are the observable behavioral outputs that can, as extreme versions, lead to impulsive and self-destructive behaviors or intense and inappropriate outbursts of anger-related behavior, which is an emotion in the sense of this paper. They might show idealizing behavior and then rapidly devaluating behavior, and this black and white splitting is emotional behavior according to the current proposed emotion model. In summary, applying the current model, BPD is a disorder rooted in a biologically vulnerable and hyper-sensitive system of subcortical affective processing. This results in dysregulated, impulsive, and often self-destructive emotions (behavioral outputs), and also leads to overwhelmingly intense, rapidly fluctuating, and often confusing feelings. This perspective emphasizes the bottom-up nature of affective dysregulation in BPD, from the earliest stages of neural processing to the subsequent behavioral responses and respective feelings.

#### 7.1.2. Other Implications

For fields relevant to User Experience (UX) and Design, the proposed emotion-model suggests going beyond explicit feedback. In designing user interfaces, software, or digital experiences, traditional methods like surveys and focus groups only capture explicit user feedback. The current model implies that the true affective impact (non-conscious liking/disliking, frustration, and engagement) occurs at a non-conscious level and is only accessible via objective technology. Understanding non-conscious affective processing is deemed more powerful in predicting actual user behavior (e.g., purchase decisions, engagement, and retention) than explicit self-reports.

Consumer neuroscience, too, can profit from the current model. In the frame of implicit attitudes, the current model directly addresses the disconnect between explicit (self-reported) and implicit (non-conscious) attitudes towards brands or products. Consumers might say they like something, but their brain’s affective processing might indicate otherwise. Since affective processing is argued to guide behavior dominantly on a basic level, measuring these non-conscious responses (e.g., via SRM, EEG, and skin conductance) can provide a more accurate prediction of consumer choices than traditional market research.

For effective advertising, understanding how certain stimuli evoke specific non-conscious affective responses allows marketers to create more potent and persuasive advertising campaigns that tap into deeper, non-verbal drivers of behavior. Similarly, brand attitude formations are heavily influenced by affective components, which neuroscientific methods can help us to understand.

Finally, applying this emotion model to non-human animals might help to provide better insight into the experienced world in organisms we cannot talk to, because the model provides simple definitions clearly separating sharp meanings of terms relevant to affection. Similarly to Occam’s razor, which was mentioned in the introduction, Morgan’s Canon is a principle in comparative psychology stating that you should not interpret an animal’s behavior as being the result of a complex, higher-level mental process (like reasoning or consciousness) if it can be adequately explained by a simpler, lower-level process (like instinct, learned associations, or trial-and-error) [71] (Anvari et al., 2025). In essence, our model offers a bridge for interpreting animal behavior in a way that aligns with Morgan’s Canon. It provides a scientific framework to explain actions via simpler, measurable brain activity or observable behavior, thus supporting the principle that we should avoid anthropomorphizing animals.

## 8. Conclusions

Addressing the problem of terminology in emotion research is crucial for advancing our understanding of how humans work. By developing a more precise and consistent language, we can improve the quality and replicability of research, facilitate interdisciplinary collaboration and communication, and ultimately gain a deeper understanding of the complex interplay between affection, cognition, and the behavior that those two important brain mechanisms guide.

In comparison to all other existing definitions of the term “emotion”, this paper suggests a completely different understanding, which arose in response to a still missing consensus that is seen as hindering further progress in the field of affective neuroscience and anything that is understood as “emotion research”. Dixon (2012) [8] mentioned that historians have long recognized the importance of keywords as both mirrors and motors of social and intellectual change [72,73] (Dixon, 2008; Williams, 1976). In particular, he wrote that “this is especially true in the realms of culture and thought, where new words, or new meanings attached to old ones, can create new concepts, and even new worldviews, which in turn transform people’s ability to imagine, experience, and understand themselves”. The current paper aims at giving the term “emotion” a new and very clear meaning, which is considered highly important.

It is herewith proposed to define “emotion” as an observable behavioral response that communicates an individual’s inner state (feeling). This means that an emotion is the outward expression—like a scared face or specific body language—that serves to signal to an observer how someone feels. It is thus further proposed to strictly separate an emotion from a feeling and from affective processing. Feelings are consciously felt bodily responses (fear is a feeling, not an emotion). They are the subjective, internal experiences that arise from affective processing, which is neural activity representing the most basic decision-making quality that guides human behavior. So, in the Walla-Emotion model, affective processing (i.e., neural activity) causes feelings (i.e., conscious bodily responses) and emotions (i.e., observable behaviors that communicate feelings). The most obvious difference to, more or less, all other understandings is that an emotion according to this model is nothing felt or experienced, it simply is understood as behavioral output.

The significance of this model is (i) the clarity of terminology (it aims to resolve the widespread ambiguity in how “affection,” “emotion,” and “feeling” are used interchangeably in scientific literature), (ii) the neurobiological basis (it emphasizes the distinct neural substrates, with subcortical areas responsible for affective processing and conscious awareness for feelings), (iii) the emphasis on communication (it highlights the social and communicative function of emotional displays (emotions as behavior)), and (iv) its implications for research by clearly separating these concepts, suggesting that researchers should use objective measures (e.g., physiological responses, brain imaging) for affective processing, and behavioral observation for emotions to complement or differentiate from subjective self-reports (for feelings). Finally, this emotion model will make any discussion about AI-driven emotion recognition very easy.

## Data Availability

Not applicable.
